# Unintentional Opioid Overdose Death Characteristics in Illinois Before and During the COVID-19 Era, 2017 to 2020

**DOI:** 10.1001/jamahealthforum.2021.3699

**Published:** 2021-11-12

**Authors:** Howard S. Kim, Joe Feinglass, Danielle M. McCarthy, Patrick M. Lank, Maryann Mason

**Affiliations:** 1 Department of Emergency Medicine, Northwestern University Feinberg School of Medicine, Chicago, Illinois; 2 Center for Health Services and Outcomes Research, Northwestern University Feinberg School of Medicine, Chicago, Illinois; 3 Associate Editor, *JAMA Network Open*, Chicago, Illinois; 4 Division of General Internal Medicine and Geriatrics, Department of Internal Medicine, Northwestern University Feinberg School of Medicine, Chicago, Illinois; 5 Toxikon Consortium, Chicago, Illinois; 6 Buehler Center for Health Policy and Economics, Northwestern University Feinberg School of Medicine, Chicago, Illinois

## Abstract

This cross-sectional study characterizes Illinois unintentional opioid overdose deaths from July 2017 through June 2020 using data from the Centers for Disease Control and Prevention State Unintentional Drug Overdose Reporting System.

## Introduction

We characterized Illinois unintentional opioid overdose deaths from July 2017 through June 2020 using the Centers for Disease Control and Prevention State Unintentional Drug Overdose Reporting System (SUDORS). Illinois SUDORS includes 42 counties, representing 91% of the state’s overdose deaths; trained abstractors enter data using death certificates, medical examiner and autopsy reports, and postmortem toxicology testing (eMethods in the Supplement). This study was approved by the Northwestern University institutional review board and adhered to the Strengthening the Reporting of Observational Studies in Epidemiology (STROBE) reporting guidelines.

We described key decedent and death scene characteristics over 6-month intervals, with attention to the January to June 2020 interval containing the pandemic start. Decedent characteristics included demographics, postmortem toxicology, and recent encounters with key touchpoints within the previous month^1^: emergency departments (EDs), hospitals, jails/prisons, and supervised residential treatment. Death scene characteristics included overdose location, bystander presence, and interventions performed.

## Results

There were 6058 opioid overdose deaths from July 2017 to June 2020. The Table summarizes decedent demographics. The Figure displays population-adjusted opioid overdose deaths over time, alongside hospitalizations for opioid overdose. Deaths and hospitalizations were stable from July 2017 to June 2019 and increased from July 2019 to June 2020.

**Table.** Decedent and Death Scene Characteristics for Unintentional Opioid Overdose Deaths in Illinois, 2017 to 2020

Characteristic	No. (%)						
	July-December 2017	2018		2019		January-June 2020	Total
		January-June	July-December	January-June	July-December		
Deaths, No	962	883	956	843	1128	1286	6058
Deaths per 100 000 population^a^	8.7	8.2	8.7	7.5	9.5	11.1	9.0
Age, mean (SD), y^b^	41.2 (12.3)	42.0 (12.6)	42.8 (12.9)	43.7 (12.5)	44.0 (12.5)	43.7 (13.2)	42.0 (12.8)
20-39	362 (37.6)	310 (35.1)	334 (34.9)	288 (34.2)	383 (34.0)	446 (34.7)	2123 (35.0)
40-59	456 (47.4)	424 (48.0)	442 (46.2)	414 (49.1)	551 (48.8)	583 (45.3)	2870 (47.4)
≥60	57 (5.9)	78 (8.8)	107 (11.2)	93 (11.0)	131 (11.6)	175 (13.6)	641 (10.6)
Sex							
Female	226 (23.5)	257 (29.1)	245 (26.5)	222 (26.3)	308 (27.3)	337 (26.2)	1595 (26.3)
Male	736 (76.5)	626 (70.9)	711 (74.4)	621 (73.7)	820 (72.7)	949 (73.8)	4463 (73.7)
Race and ethnicity^c^							
Black	216	222 (25.1)	306 (32.0)	255 (30.2)	390 (34.6)	471 (36.6)	1860 (30.7)
Hispanic	84	93 (10.5)	108 (11.3)	98 (11.6)	109 (9.7)	157 (12.2)	638 (10.5)
White	496	508 (57.5)	509 (53.2)	464 (55.0)	604 (53.5)	624 (48.5)	3205 (52.9)
Missing	155	53 (6.0)	25 (2.6)	22 (2.6)	12 (1.1)	17 (1.3)	284 (4.7)
Other^d^	11	7 (0.8)	8 (0.8)	4 (0.5)	13 (1.2)	17 (1.3)	60 (1.0)
Previous overdose ever	114 (12.0)	107 (12.1)	97 (10.2)	95 (11.3)	132 (11.7)	110 (8.6)	655 (10.8)
Any recent touchpoint encounter within 1 mo^e^	NA	NA	NA	NA	133 (11.8)	174 (13.5)	NA
From ED visit	NA	NA	NA	NA	57 (5.1)	72 (5.6)	NA
From jail or prison	31 (3.2)	35 (4.0)	31 (3.2)	41 (4.9)	27 (2.4)	42 (3.3)	207 (3.4)
From hospital	17 (1.8)	32 (3.6)	32 (3.3)	41 (4.9)	63 (5.6)	76 (5.9)	261 (4.3)
From supervised SUD residential facility	20 (2.1)	14 (1.6)	19 (2.0)	18 (2.1)	26 (2.3)	24 (1.9)	121 (2.0)
OUD treatment							
Current	49 (5.1)	52 (5.9)	34 (3.6)	39 (4.6)	63 (5.6)	52 (4.0)	289 (4.8)
Ever	146 (15.2)	131 (14.8)	137 (14.3)	115 (13.6)	162 (14.4)	152 (11.0)	843 (13.9)
Recent relapse	75 (7.8)	72 (8.2)	74 (7.7)	69 (8.2)	57 (5.1)	57 (4.4)	655 (10.8)
Own home overdose	585 (60.8)	520 (58.9)	570 (59.6)	512 (60.7)	678 (60.1)	787 (61.2)	3652 (60.3)
EMS at scene	895 (93.0)	824 (93.3)	906 (94.8)	798 (94.7)	1062 (94.1)	1208 (93.9)	5693 (94.0)
Transported to ED	216 (22.5)	228 (25.8)	268 (28.0)	233 (27.6)	319 (28.3)	306 (23.8)	1570 (25.9)
Bystander present	192 (20.0)	262 (29.7)	355 (37.1)	350 (41.5)	573 (50.8)	647 (50.3)	2379 (39.3)
Bystander relationship^f^							
Family	NA	NA	NA	NA	181 (31.6)	215 (33.2)	NA
Friend	NA	NA	NA	NA	112 (19.5)	146 (22.6)	NA
Partner	NA	NA	NA	NA	152 (26.5)	166 (25.7)	NA
Roommate	NA	NA	NA	NA	35 (6.1)	58 (9.0)	NA
Stranger	NA	NA	NA	NA	51 (8.9)	40 (6.2)	NA
User	NA	NA	NA	NA	30 (5.2)	37 (5.7)	NA
Bystander CPR^f^	NA	NA	NA	NA	92 (16.1)	93 (14.4)	NA
Naloxone administered	228 (23.7)	279 (31.6)	325 (34.0)	312 (31.2)	449 (34.0)	465 (31.8)	2058 (34.0)
Who administered naloxone^f^							
Bystander	NA	NA	NA	NA	23 (5.1)	20 (4.3)	NA
EMS	NA	NA	NA	NA	167 (37.2)	172 (37.0)	NA
Law enforcement	NA	NA	NA	NA	28 (6.2)	39 (8.4)	NA
Hospital	NA	NA	NA	NA	33 (7.3)	21 (4.5)	NA
Unknown	NA	NA	NA	NA	178 (39.6)	195 (41.9)	NA
Fentanyl positive	585 (60.8)	599 (67.8)	722 (75.5)	649 (77.0)	873 (77.4)	1056 (82.1)	4484 (74.0)
Prescription opioid positive	NA	NA	NA	175 (20.8)	258 (22.9)	288 (22.4)	NA

Abbreviations: CPR, cardiopulmonary resuscitation; ED, emergency department; EMS, emergency medical services; NA, not available; OUD, opioid use disorder; SUD, substance use disorder.

^a^ Population-adjusted deaths include only those deaths among Illinois residents, given that the population denominators were sourced from US Census Bureau annual estimates of the Illinois resident population. The numbers of confirmed Illinois resident deaths per period were 915, 833, 901, 799, 1065, and 1241, respectively.

^b^ Decedents younger than 20 years were censored because the total number per period did not exceed the minimum requirement for deidentified reporting.

^c^ Percentages not listed for July to December 2017 owing to high missingness (16.1%).

^d^ Other includes the following non-Hispanic categories: American Indian or Alaska Native, Asian or Pacific Islander, and Other (unspecified).

^e^ Some variables were introduced into the Centers for Disease Control and Prevention State Unintentional Drug Overdose Reporting System in 2019 and are therefore not available in earlier periods.

^f^ Bystander relationship, bystander CPR, and who administered naloxone percentage denominators are bystanders present, bystanders present, and naloxone administered, respectively.

**Figure.**
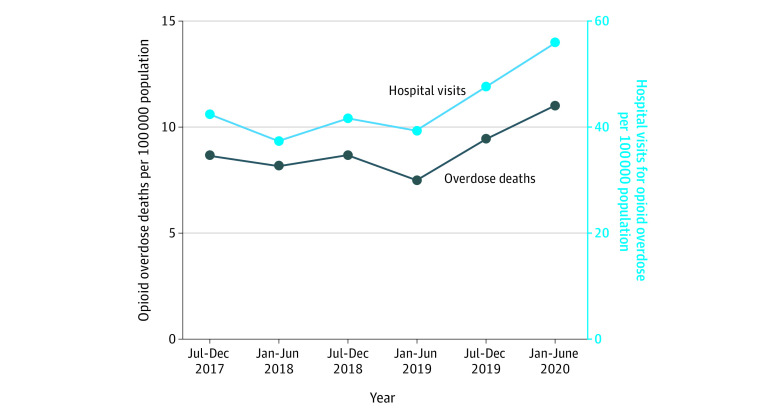
Population-Adjusted Illinois Opioid Overdose Deaths and Hospital Visits for Opioid Overdose Opioid overdose death data are from the Centers for Disease Control and Prevention State Unintentional Drug Overdose Reporting System. Hospital visit data are from the Illinois Hospital Association Comparative Healthcare and Hospital Data Reporting services database, which represents 214 nonfederal Illinois hospitals. Hospital visits include discharged emergency department visits and hospitalizations with a primary *International Statistical Classification of Diseases and Related Health Problems, Tenth Revision* diagnosis of opioid overdose.

Decedents were increasingly Black and Hispanic over time and exceeded White decedents by January to June 2020; decedents were also increasingly aged 60 years or older. In January to June 2020, 174 (13.5%) had a recent touchpoint encounter compared with 133 (11.8%) in July to December 2019. Toxicology testing results were increasingly positive for fentanyl, reaching 1056 (82.1%) by January to June 2020; prescription opioid positivity remained stable.

During the January to June 2020 period coinciding with COVID-19, most overdose deaths occurred in the decedent’s home (787 [61.2%]) with bystanders present (647 [50.3%]), similar to preceding periods. Some death scene characteristics were marginally lower in January to June 2020 compared with July to December 2019: bystander cardiopulmonary resuscitation (93 [14.4%] vs 92 [16.1%]), ED transportation (306 [23.8%] vs 319 [28.3%]), and naloxone administration (465 [31.8%] vs 449 [34.0%])—although these rates were not dissimilar to earlier periods. Several decedent characteristics relating to previous opioid use disorder (OUD) reached their lowest rate in January to June 2020, such as any previous overdose (110 [8.6%]) or OUD treatment (152 [11.0%]), despite higher rates of recent touchpoint encounters.

## Discussion

Several study findings warrant further discussion. First, the proportion of Black, Hispanic, and older adult decedents has continued to increase. It is imperative that we address known racial disparities in access to buprenorphine and linkage to treatment after nonfatal overdose^2^; we must also devote attention to identifying OUD among older adults. Second, fentanyl is now pervasive in the drug supply and far more prevalent in postmortem toxicology results than prescription opioids. Greater resources should be devoted to the provision of fentanyl test strips and take-home naloxone, particularly from key touchpoints encountered by 1 in 7 decedents in this study. Third, several indicators of previous OUD nadired in the COVID-19 period, despite higher rates of recent touchpoint encounters. Harm reduction initiatives, such as naloxone distribution, should more broadly target individuals without a history of prior overdose. Finally, most overdoses occur in a decedent’s home and with bystanders present. Future interventions should equip and empower persons who use opioids and their surrogates to use opioids safely in communion and with resources to reverse overdose.

These findings are limited to a single state and rely on death certificate and medical examiner reports, which may be incomplete. However, they serve as the first characterization of decedent characteristics during the COVID-19 era and demonstrate that opioid overdose deaths began to increase in late 2019 in Illinois with decedent characteristics largely continuing existing trends. These data may inform missed opportunities for overdose prevention and development of carefully tailored harm reduction policies.

## Notes

Supplement.
eMethods
