# Supplementary material for: Unintentional Opioid Overdose Death Characteristics in Illinois Before and During the COVID-19 Era, 2017 to 2020
Source: JAMA Health Forum. 2021 Nov 12;2(11):e213699. doi: 10.1001/jamahealthforum.2021.3699 (PMC8796894; doi:10.1001/jamahealthforum.2021.3699)
Supplement: Supplement. — eMethods [file jamahealthforum-e213699-s001.pdf]

Supplemental Online Content

Kim HS, Feinglass J, McCarthy DM, et al. Unintentional opioid overdose death characteristics in Illinois before and during the COVID-19 era, 2017 to 2020. *JAMA Health Forum*. 2021;2(11):e213699. doi:10.1001/jamahealthforum.2021.3699

eMethods.

This supplemental material has been provided by the authors to give readers additional information about their work.

eMethods:

I. Illinois Statutory Provisions Relevant to Naloxone

Illinois enacted broad statutory protections for bystanders to possess and use naloxone without a prescription in September 2015 (IL Public Act 099-0480; the Heroin Crisis Act). This authorized a standing order for retail pharmacists to dispense naloxone without a prescription, clarified that registered community Drug Overdose Prevention Programs could distribute naloxone to persons who may be in a position to reverse an overdose, provided criminal liability protection to health care professionals who prescribe or dispense naloxone in good faith, and provided civil liability protection to individuals who administer naloxone to reverse an overdose.

© 2021 Kim HS, et al. *JAMA Health Forum.*

II. SUDORS variable definitions

Variable definitions and coding instructions are set by the Centers for Disease Control and Prevention. Individual state grantees perform primary data abstraction and send coded data to the CDC for cleaning. The Illinois Department of Public Health maintains the Illinois data. CDC updates the state datasets periodically. We utilized the April 22, 2021 release of IL SUDORS data.

Race/Ethnicity: Decedent race and ethnicity is abstracted from the death certificate, but if this information is missing from the death certificate it is abstracted from the coroner/medical examiner's report.

Bystander: A bystander is an individual who was physically nearby either during or shortly preceding a drug overdose who potentially had an opportunity to intervene and respond to the overdose. First responders or medical professionals called to the scene are not considered bystanders. Because a bystander must be an individual with an opportunity to intervene, a cutoff at the minimum age of 11 years old is used.

Naloxone Administered: Indicates whether the decedent was administered naloxone by a layperson, EMS, police, fire, or ED/hospital personnel. This information can be gathered from narrative reports of the overdose scene, medication administration forms, or post-mortem toxicology testing. For narrative reports, there must be evidence that naloxone was actually administered to the patient (i.e., discovery of used naloxone packaging is insufficient if there is no documentation of administration to the decedent). For post-mortem toxicology testing, if both naloxone and buprenorphine are detected, the decedent is not considered to have received naloxone due to the possibility of buprenorphine/naloxone use.

Own Home Overdose: Indicates whether the overdose occurred in the decedent's own home.

Fentanyl Positivity: Detection of the following items is considered positive for fentanyl: fentanyl, norfentanyl, despropionylfentanyl, 3-methylfentanyl, 3-methylthiofentanyl, 4-fluorobutyrylfental, 4-fluorofentanyl, 4-fluoroisobutyrylfentayl, 4-methyoxybutyrfentanyl, carfentanil, crotonylfentanyl, cyclopentylfentanyl, cycloropylfentanyl, furanylethylfentanyl, furanylfetanyl,acetnylfentanyl, acrylfetanyl, alfentanil, benzylfetanyl, beta-hydroxythiofentanyl, butyrylfentanyl, isobutyrylfentanyl, methoxyacetylfentanyl, oxfentanil, sufentanil, tetrahydrofuranylfentanyl, valerylfentanyl, despropionylfentanyl, N-phenethyl-4-piperidone, U-47700, U-48800, U-49900, U-51754, AH-7921, MT-45

© 2021 Kim HS, et al. *JAMA Health Forum.*

III. Population-Adjusted Rates

We obtained Illinois population data for each year of interest from the U.S. Census Bureau, Population Division report of “Annual Estimates of the Resident Population for Counties in Illinois, April 1, 2010 to July 1, 2019” (Release Date March 2020). As of September 23, 2021, year 2020 data are not available “because of impacts of the COVID-19 pandemic on data collection.” We therefore used 2019 population data to calculate 2020 population-adjusted figures.

Opioid overdose death rates: We included only opioid deaths occurring among Illinois residents (i.e., excluding opioid overdose deaths from non-Illinois residents), given that the denominator comes from U.S. Census Bureau annual estimates of the Illinois resident population. Because SUDORS does not contain data from all Illinois counties, we used only the population data from counties contributing data to SUDORS to calculate population-adjusted overdose death rates.

Hospital visits for opioid overdose: We used Illinois state population data to calculate population-adjusted rates of hospitals visits for opioid overdose in the Illinois Hospital Association Comparative Healthcare and Hospital Data Reporting Services database.

© 2021 Kim HS, et al. *JAMA Health Forum.*
